# Positive faecal immunochemical test predicts the onset of inflammatory bowel disease: A nationwide, propensity score-matched study

**DOI:** 10.3389/fimmu.2023.1128736

**Published:** 2023-02-13

**Authors:** Eunyoung Lee, Gil Ho Lee, Bumhee Park, Sung Soo Ahn, Choong-Kyun Noh

**Affiliations:** ^1^ Department of Biomedical Informatics, Ajou University School of Medicine, Suwon, Republic of Korea; ^2^ Office of Biostatistics, Ajou Research Institute for Innovative Medicine, Ajou University Medical Center, Suwon, Republic of Korea; ^3^ Department of Gastroenterology, Ajou University School of Medicine, Suwon, Republic of Korea; ^4^ Division of Rheumatology, Department of Internal Medicine, Yongin Severance Hospital, Yonsei University College of Medicine, Yongin, Republic of Korea

**Keywords:** faecal immunochemical test, inflammatory bowel disease, incidence, prediction, risk

## Abstract

**Background & aims:**

The faecal immunochemical test (FIT), a non-invasive test for screening colorectal cancer (CRC), is being increasingly understood to reflect heightened inflammation. We aimed to investigate the association between abnormal FIT results and onset of inflammatory bowel disease (IBD), a disease characterized with chronic gut mucosal inflammation.

**Methods:**

Participants in the Korean National Cancer Screening Program for CRC between 2009–2013 were analysed and divided into positive and negative FIT result groups. The incidence rates of IBD after screening were calculated after excluding cases of haemorrhoids, CRC, and IBD at baseline. Cox proportional hazard analyses were used to identify independent risk factors for IBD occurrence during follow-up, and 1:2 propensity score matching was performed as a sensitivity analysis.

**Results:**

In total, 229,594 and 815,361 participants were assigned to the positive and negative FIT result groups, respectively. The age- and sex-adjusted incidence rates of IBD in participants with positive and negative test results were 1.72 and 0.50 per 10,000 person-years, respectively. Adjusted Cox analysis revealed that FIT positivity was associated with a significantly higher risk of IBD (hazard ratio 2.93, 95% confidence interval: 2.46, 3.47, P <.001), which was consistent for both disease subtypes of ulcerative colitis and Crohn’s disease. The results of Kaplan–Meier analysis in the matched population yielded identical findings.

**Conclusions:**

Abnormal FIT results could be a preceding sign of incident IBD in the general population. Those with positive FIT results and suspected IBD symptoms could benefit from regular screening for early disease detection.

## Introduction

Inflammatory bowel disease (IBD) is a chronic, potentially life-threatening disorder that affects the digestive system and presents with recurrent episodes of abdominal pain, diarrhoea, haematochezia, fever, and weight loss (1). According to the inflamed regions, their pattern, and pathologic findings in the gastrointestinal tract, IBD is classified into two different diseases: ulcerative colitis (UC) and Crohn’s disease (CD). The underlying pathogenesis of IBD involves a complex interplay of factors, including dysregulation of intestinal microbiota, host genetic susceptibility, and environmental triggers, resulting in an immunological imbalance, which remains largely unknown (2–4). While IBD has been traditionally considered more common in Western countries, a continuous rise in the incidence of IBD has been reported in recent years, especially in Asia (5, 6). Therefore, there is a need for diagnostic tests that are useful for early detection of IBD.

Immunologic perturbation, particularly in the adaptive immune system, is thought to be a crucial element responsible for the impairment of bowel equilibrium and causing chronic gut inflammation in IBD (7–9). Consequently, mucosal inflammation documented by endoscopy or imaging of the gastrointestinal tract is a typical finding in patients with IBD (10). The faecal immunochemical test (FIT) is a non-invasive test that measures faecal haemoglobin concentrations using an antibody specific for human haemoglobin, and has been widely used for colorectal cancer (CRC) screening by detecting blood in the faeces (11, 12); however, since a characteristic feature observed in IBD is the presence of mucosal injury, it is possible that a positive FIT may be an early sign of IBD (13). Additionally, growing evidence suggests that abnormal FIT results, without a definite focus on bleeding, may reflect underlying systemic inflammation and could be correlated with chronic inflammatory diseases (14). However, the association between positive FIT results and incident IBD has not yet been determined in the general population. Because of this, we aimed to evaluate whether abnormalities of the gut mucosa, defined as a positive FIT result, are associated with the development of IBD, and to evaluate the risk factors in those who participated in the national program for CRC screening.

## Materials and methods

### Data source

The Korean National Cancer Screening Program (KNCSP) is a national program operated by the South Korean government and is designed to screen for cancers in the stomach, liver, colorectum, breast, and cervix according to specific recommendations (15). The results of the KNCSP are stored in the National Health Insurance Sharing Service-National Health Information Database (NHIS-NHID); the use of this data is approved for authorised researchers. In South Korea, the NHIS provides medical services covered by the national health insurance for >50 million individuals (approximately 97% of the entire population) (16).

In the KNCSP for screening colorectal cancer (CRC), the government provides an annual faecal immunochemical test (FIT) for individuals aged ≥50 years to screen for CRC. In addition, for those with positive FIT results, the NHIS provides subsequent examinations by either double-contrast barium enema or colonoscopy, based on the preference of the individual. In this study, data of the population who participated in the KNCSP for CRC between 2009–2013, including data from the NHIS, were utilised. and participants were followed up until December 21, 2019. Details of the study design, participants, and data acquisition have been described previously (17). This study was approved by the institutional review board of Ajou University Hospital (approval No. AJIRB-MED-EXP-20-479). The requirement for obtaining individual informed consent was waived because the entire dataset was anonymised.

### Study design and selection of eligible participants

In total, 9,161,668 subjects participated in the KNCSP for CRC between the year of 2009–2013. Among them, those who did not undergo a FIT, had a history of colorectal cancer, and had immune-mediated inflammatory diseases (IBD, rheumatoid arthritis, psoriatic arthritis, and systemic lupus erythematosus) that could influence FIT results were excluded. Of the 8.646,887 participants, FIT positive (FIT [+]) and FIT-negative (FIT [−]) groups were separated by applying a 1:1 matched random sampling according to age and sex.

**Figure 1:**
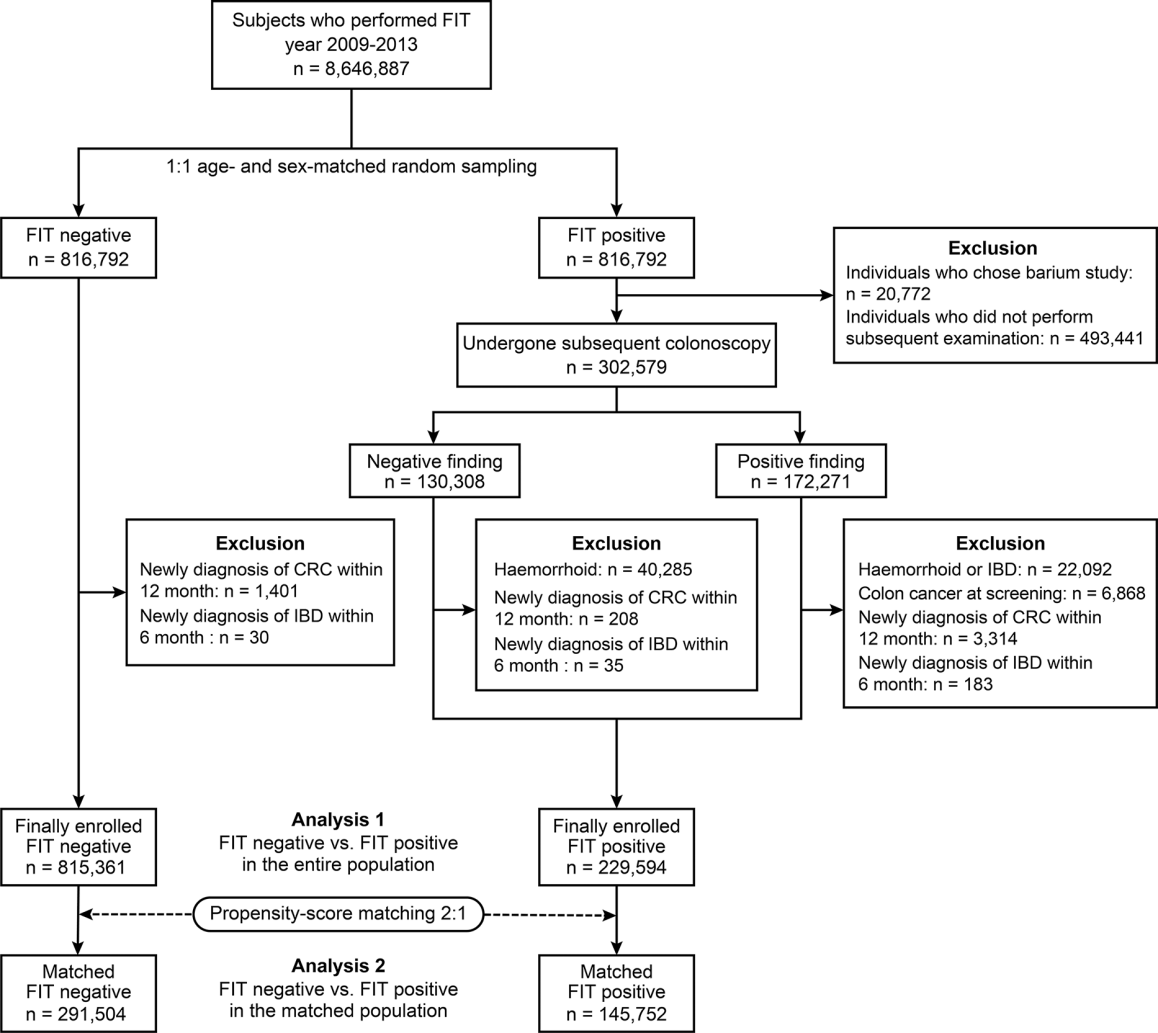
A Flow Diagram of Selecting the FIT (+) and FIT (-) Group. FIT, faecal immunochemical test; CRC, colorectal cancer; IBD, inflammatory bowel disease.

In the FIT (+) group, participants who had undergone colonoscopy as a subsequent evaluation were selected, and colonoscopy findings were categorised according to reports submitted to the KNCSP. Colonoscopy-positive findings were defined as documented gross abnormal mucosal lesions of suspected colon cancer, colon cancer, polyps, diverticulosis, and inflammatory lesions. We excluded subjects diagnosed with haemorrhoids, IBD, or CRC according to the colonoscopy results. In addition, those who were diagnosed with IBD and CRC within 6 and 12 months after undergoing a FIT, based on the tenth revision codes of the International Statistical Classification of Diseases (ICD-10 codes), were excluded, as they could be a missed IBD and CRC (18). In the FIT (−) group, those diagnosed with IBD within 6 months and CRC within 12 months after screening were also excluded (**Figure 1**).

### Faecal immunochemical tests of the participants

Faecal samples from subjects participating in the KNCSP for CRC were collected according to the general instructions provided, and were sent to an assigned centre for analyses, which were reported as positive or negative. Faecal immunochemical test assessment was conducted using qualitative and quantitative methods. For the qualitative method, a commercially available kit was used according to the cut-off values provided in the kit as follows: FOBtest, Humasis Co., Korea (50 ng/mL [10 ug/g]), SD Bioline FOB, SD Co., Korea (30 ng/mL [6 ug/g]), ASAN Easy Test FOB, Asan Pharm Co., Korea (50 ng/mL [10 ug/g]), and OC-Hemocatch Lignt™, Eiken Chemical Co., Japan (50 ng/mL [10 ug/g]). The faecal haemoglobin value was determined by latex agglutination nephelometric immunoassay in a quantitative assay (Eiken Chemical Co.), in which the cut-off value of the corresponding institution was also reported (19, 20).

### Covariates

Those who participated in the CRC screening program completed questionnaires on smoking, alcohol drinking, and physical exercise, and submitted them to the responsible institutions. In addition, data on age, sex, anthropometric measurements including weight, height, and body mass index (BMI), medical and family history, socioeconomic status, and clinical information were collected. The variables used in our analysis were as follows: sex; age; BMI; smoking status (no or yes); alcohol consumption (no or yes); insurance type (medical aid or national health insurance); comorbidities (hypertension, diabetes, or dyslipidaemia); and laboratory results of haemoglobin, total cholesterol, triglyceride, high-density lipoprotein (HDL) cholesterol, low-density lipoprotein (LDL) cholesterol, aspartate aminotransferase (AST), and alanine aminotransferase (ALT). The normal values of laboratory data were set according to the predefined cut-off values: haemoglobin (male: ≥13 g/dL, female: ≥12 g/dL), total cholesterol (<200 mg/dL), triglyceride (<150 mg/dL), HDL cholesterol (≥60 mg/dL), LDL-cholesterol (<130 mg/dL), AST (≤40 IU/L), and ALT (≤35 IU/L) (21).

### Definition of inflammatory bowel disease and CRC

The outcome of interest was the incidence of inflammatory bowel disease (IBD), including ulcerative colitis (UC) and Crohn’s disease (CD). In South Korea, when an individual utilises a medical service covered by national insurance, all medical institutions must provide a record of their healthcare usage, which is collected in the National Health Insurance Sharing Service-National Health Information Database (NHIS-NHID) containing diagnosis codes (22). To search for cases of IBD and CRC, we first identified CRC and IBD using primary and secondary codes of the ICD-10 codes (C18–20 for CRC and K50–51 for IBD). Furthermore, as the Korean government grants special exemption codes for patients with rare and intractable diseases to subsidise their healthcare expenses for those fulfilling the criteria defined by the National Health Insurance, we used a special exemption code (V code) to ensure an accurate diagnosis of IBD. Thus, in this study, IBD cases that were designated with a special exemption code of V130 (CD) and V131 (UC) were selected (23).

### Statistical analysis

Continuous and categorical variables were presented as a mean (SD) and a number (frequency), respectively. Differences between groups were evaluated using Student’s t-test and chi-squared test for continuous and categorical variables. The follow-up duration was set as the date of initial screening to the diagnosis date of IBD in those diagnosed as IBD, whereas it was defined as the last follow-up in those who did not develop IBD. Crude and age-and sex-adjusted incidence rates per 10,000 person-years were calculated using the number of incident IBD cases and person-time of those at risk. Furthermore, Cox proportional hazard analyses were used to identify independent risk factors for IBD during follow-up. As a sensitivity analysis, 1:2 propensity score matching was performed in order to adjust for the differences in baseline characteristics. Kaplan–Meier analysis and the log-rank test were used to compare the differences in IBD incidence. Statistical analyses were conducted using SAS statistical software (SAS Institute, Cary, NC, USA), and a two-tailed P <.05 was considered significant.

## Results

### Comparison of baseline characteristics between the FIT (+) and FIT (−) groups

Among the 1,044,955 participants, 815,361 and 229,594 individuals were divided into FIT (−) and FIT (+) groups, respectively (**Table 1**). Of the total number of participants, 565,280 (54.10%) were male, the overall mean (SD) age was 62.06 (8.58) years, and the proportion of those aged 50–59 years was the highest, accounting for 43.77% of the participants. In addition, the proportions of current smokers and alcohol drinkers in the total population were 16.1% and 21.13%, respectively. There were significant differences between the FIT (−) and FIT (+) groups in the baseline characteristics investigated, including demographic data and laboratory results, with the exception of triglyceride levels (P = .490).

**Table 1:** Clinical and laboratory characteristics of the participants at baseline.

	Total cohort (n = 1,044,955)	FIT (−) group (n = 815,361)	FIT (+) group (n = 229,594)	*P*-value
Demographic data				
Sex				<.001
Male	565,280 (54.10)	436,092 (53.48)	129,188 (56.27)	
Female	479,675 (45.90)	379,269 (46.52)	100,406 (43.73)	
Age at screening, years	62.06 ± 8.58	62.4 ± 8.73	60.86 ± 7.90	<.001
50–59	457,331 (43.77)	346,316 (42.47)	111,015 (48.35)	<.001
60–69	352,523 (33.74)	273,036 (33.49)	79,487 (34.62)	
70–79	203,606 (19.48)	167,777 (20.58)	35,829 (15.61)	
≥80	31,495 (3.01)	28,232 (3.46)	3,263 (1.42)	
BMI, kg/m^2^	24.14 ± 3.03	24.10 ± 3.03	24.27 ± 2.99	<.001
Smoking status				<.001
No	754,426 (83.86)	592,256 (83.99)	162,170 (83.38)	
Yes	145,186 (16.14)	112,858 (16.01)	32,328 (16.62)	
Alcohol drinking				<.001
No	709,427 (78.87)	559,665 (79.38)	149,762 (77.01)	
Yes	190,055 (21.13)	145,337 (20.62)	44,718 (22.99)	
Insurance type				<.001
Medical aid	44,650 (4.27)	35,631 (4.37)	9,019 (3.93)	
National health insurance	100,265 (95.73)	779,696 (95.63)	220,569 (96.07)	
Underlying comorbidity				
Hypertension				<.001
No	656,727 (87.75)	518,754 (93.40)	137,973 (92.47)	
Yes	47,872 (12.25)	36,644 (6.60)	11,228 (7.53)	
Diabetes mellitus				<.001
No	656,727 (93.21)	518,754 (93.40)	137,973 (92.47)	
Yes	47,872 (6.79)	36,644 (6.60)	11,228 (7.53)	
Dyslipidaemia				<.001
No	501,128 (67.06)	397,006 (67.49)	104,122 (65.44)	
Yes	246,187 (32.94)	191,200 (32.51)	54,987 (34.56)	
Laboratory results				
Haemoglobin, g/dL	13.84 ± 1.49	13.85 ± 1.49	13.79 ± 1.50	<.001
Total cholesterol, mg/dL	198.72 ± 40.78	198.30 ± 40.43	200.10 ± 42.02	<.001
Triglyceride, mg/dL	139.19 ± 97.69	139.20 ± 98.12	139.30 ± 96.11	.490
HDL-cholesterol, mg/dL	54.11 ± 24.20	54.08 ± 24.40	54.23 ± 23.46	.010
LDL-cholesterol, mg/dL	118.27 ± 48.35	117.90 ± 47.51	119.50 ± 51.25	<.001
AST, IU/L	27.22 ± 19.77	26.78 ± 19.10	28.81 ± 21.94	<.001
ALT, IU/L	25.34 ± 24.51	24.87 ± 22.24	27.05 ± 31.33	<.001

Data are presented as mean (SD) or number (%).

FIT, faecal immunochemical test; BMI, body mass index; HDL, high-density lipoprotein; LDL, low-density lipoprotein; AST, aspartate aminotransferase; ALT, alanine aminotransferase.

### Incidence of IBD during the follow-up period according to FIT positivity

**Figure 2:**
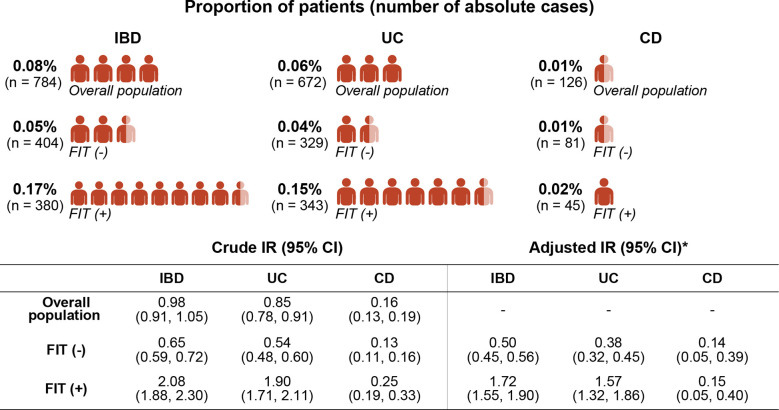
Comparison of IBD incidence rates between the FIT (−) and FIT (+) groups. *Values adjusted for age and sex. IBD, inflammatory bowel disease; FIT, faecal immunochemical test; UC, ulcerative colitis; CD, Crohn’s disease; IR, incidence rate per 10,000 person-years.

**Figure 3:**
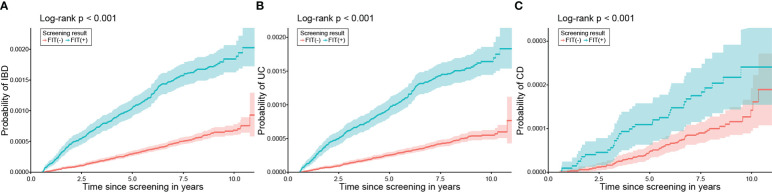
Cumulative incidence of IBD, UC, and CD according to FIT results in those with normal haemoglobin. The incidences of **(A)** IBD, **(B)** UC, and **(C)** CD were significantly higher in the positive FIT group than in the negative FIT group. IBD, inflammatory bowel disease; UC, ulcerative colitis; CD, Crohn’s disease; FIT, faecal immunochemical test.

A total of 784 participants (incidence rates [IR] 0.98/10,000 person-years [PY]) were diagnosed with IBD during a mean follow-up period of 7.59 years (SD 1.81). Among those who developed IBDs, 672 (IR 0.85/10,000 PY) and 126 (IR 0.16/10,000 PY) were diagnosed with UC and CD, respectively, and 14 patients were diagnosed with both UC and CD. The incidence of UC and CD was higher in the FIT (+) group than in the FIT (−) group, even after adjusting for age and sex (**Figure 2**). Moreover, the cumulative incidence of IBD in the FIT (+) group was also significantly higher than that in the FIT (−) group in a Kaplan–Meier analysis among subjects with normal haemoglobin values (all P <.001) (**Figure 3**).

### The occurrence of IBD according to different time intervals, sex, and age

When the incidence of IBD was categorised according to three different time intervals of <2 years, 2–5 year, and ≥5 years, the incidence of IBD was observed to be the highest within the second year of screening (IR 0.82/10,000 PY, 95% CI: 0.70, 0.95), which was not affected by disease subtypes. In addition, a trend of decreasing incidence of IBD after screening was demonstrated for both UC and CD. In particular, those in the FIT (+) group were more frequently diagnosed with IBD, as well as UC, during follow-up in the adjusted analyses, but this was not evident in CD (**Table 2**).

**Table 2:** Incidence rates of IBD categorized by time intervals after screening and disease subgroups.

Groups	Number of incident cases			Crude IR (95% CI)			Adjusted IR (95% CI)^a^		
	<2 year	2–5 years	≥5 years	<2 year	2–5 years	≥5 years	<2 year	2–5 years	≥5 years
IBD (n = 784)									
Overall population	169	310	305	0.82 (0.70, 0.95)	0.61 (0.55, 0.68)	0.39 (0.35, 0.44)	–	–	–
FIT (−) group	69	164	171	0.43 (0.34, 0.54)	0.41 (0.36, 0.48)	0.28 (0.25, 0.33)	0.36 (0.13, 0.99)	0.36 (0.13, 0.97)	0.21 (0.11, 0.43)
FIT (+) group	100	146	134	2.19 (1.80, 2.67)	1.30 (1.10, 1.53)	0.75 (0.64, 0.89)	2.09 (0.76, 5.72)	0.58 (0.22, 1.58)	0.58 (0.29, 1.17)
UC (n = 672)									
Overall population	151	267	254	0.73 (0.62, 0.85)	0.53 (0.47, 0.59)	0.33 (0.29, 0.37)	–	–	–
FIT (−) group	61	135	133	0.38 (0.29, 0.49)	0.34 (0.29, 0.40)	0.22 (0.19, 0.26)	0.33 (0.12, 0.93)	0.25 (0.07, 0.90)	0.15 (0.05, 0.43)
FIT (+) group	90	132	121	1.97 (1.60, 2.42)	1.17 (0.99, 1.39)	0.68 (0.57, 0.81)	1.82 (0.66, 5.05)	0.47 (0.13, 1.65)	0.53 (0.19, 1.48)
CD (n = 126)									
Overall population	18	48	60	0.09 (0.05, 0.14)	0.09 (0.07, 0.13)	0.08 (0.06, 0.10)	–	–	–
FIT (−) group	8	31	42	0.05 (0.02, 0.10)	0.08 (0.06, 0.11)	0.07 (0.05, 0.09)	n/a^b^	0.09 (0.02, 0.31)	0.07 (0.02, 0.25)
FIT (+) group	10	17	18	0.22 (0.12, 0.41)	0.15 (0.09, 0.24)	0.10 (0.06, 0.16)	n/a^b^	0.09 (0.02, 0.30)	0.06 (0.02, 0.19)

^a^Values adjusted for age and sex.

^b^Values are incalculable because of the small number of patients.

IBD, inflammatory bowel disease; IR, incidence rate per 10,000 person-years; UC, ulcerative colitis; CD, Crohn’s disease; FIT, faecal immunochemical test; n/a, not applicable.

Analysis of the incidence of IBD based on sex and age showed that the overall incidence was the highest in those aged 50–59 years in both sexes (IR 1.29/10,000 PY for males and 0.83/10,000 PY for females), which decreased gradually with age. For disease subtypes, UC was most common in the age group of 50–59 years, CD was most frequently diagnosed in the age group of 60–69 years, and the incidence of UC and CD was consistently higher in males than in females across all age groups (**Table 3**).

**Table 3:** Incidence of IBD classified according to sex and age.

	IBD	UC	CD
Male			
Age at screening, years			
50–59	1.29 (1.17, 1.43)	1.19 (1.01, 1.39)	0.15 (0.13, 0.18)
60–69	1.21 (1.10, 1.34)	1.05 (0.90, 1.23)	0.19 (0.16, 0.22)
70–79	0.85 (0.77, 0.94)	0.70 (0.60, 0.81)	0.16 (0.14, 0.19)
≥80	0.51 (0.46, 0.56)	0.40 (0.34, 0.47)	0.11 (0.09, 0.13)
Female			
Age at screening, years			
50–59	0.83 (0.75, 0.92)	0.70 (0.60, 0.82)	0.14 (0.12, 0.16)
60–69	0.78 (0.70, 0.86)	0.62 (0.53, 0.72)	0.16 (0.14, 0.19)
70–79	0.54 (0.49, 0.60)	0.41 (0.35, 0.48)	0.14 (0.12, 0.17)
≥80	0.33 (0.29, 0.36)	0.24 (0.20, 0.27)	0.09 (0.08, 0.11)

The values presented indicate IR and 95% CI.

IBD, inflammatory bowel disease; UC, ulcerative colitis; CD, Crohn’s disease; IR, incidence rate per 10,000 person-years.

### Factors associated with the occurrence of IBD

The Cox proportional hazard analysis indicated that a positive FIT result (hazard ratio [HR] 2.93, 95% CI: 2.46, 3.47, P <.001), male sex (HR 1.64, 95% CI: 1.35, 2.00, P <.001), and abnormal high-density lipoprotein (HDL) cholesterol level (HR 1.40, 95% CI: 1.09, 1.81, P = .010) increased the risk of developing IBD, while an increase in body mass index (BMI; HR 0.92, 95% CI: 0.89, 0.95, P <.001) and diabetes mellitus (HR 0.63, 95% CI: 0.40, 1.00, P = .050) had a negative association with the incidence of IBD (**Table 4**).

**Table 4:** Independent predictors of developing IBD during the follow-up.

	IBD		UC		CD	
	HR (95% CI)^a^	*P*-value	HR (95% CI)^a^	*P*-value	HR (95% CI)^a^	*P*-value
Screening result						
FIT (−)	Ref		Ref		Ref	
FIT (+)	2.93 (2.46, 3.47)	<.001	3.12 (2.60, 3.76)	<.001	2.04 (1.31, 3.19)	.002
Sex						
Female	Ref		Ref		Ref	
Male	1.64 (1.35, 2.00)	<.001	1.79 (1.45, 2.22)	<.001	1.07 (0.66, 1.75)	.782
Age at screening, years						
50–59	2.67 (0.99, 7.17)	.052	3.12 (1.00, 9.75)	.051	1.07 (0.66, 1.75)	.782
60–69	2.46 (0.91, 6.62)	.076	2.72 (0.87, 8.52)	.087	1.53 (0.21, 11.21)	.677
70–79	1.83 (0.67, 5.03)	.241	1.95 (0.61, 6.25)	.260	1.74 (0.24, 12.82)	.586
≥80	Ref		Ref		Ref	
BMI	0.92 (0.89, 0.95)	<.001	0.92 (0.89, 0.95)	<.001	0.96 (0.89, 1.04)	.334
Smoking status						
No	Ref		Ref		Ref	
Yes	0.96 (0.75, 1.22)	.740	0.85 (0.65, 1.11)	.237	1.68 (0.94, 2.98)	.078
Alcohol drinking						
No	Ref		Ref		Ref	
Yes	0.86 (0.68, 1.08)	.181	0.90 (0.70, 1.14)	.375	0.73 (0.40, 1.35)	.319
Insurance type						
Medical aid	Ref		Ref		n/a^b^	
National health insurance	0.58 (0.24, 1.4)	.228	0.49 (0.20, 1.18)	.112	n/a^b^	
Hypertension						
No	Ref		Ref		Ref	
Yes	0.91 (0.67, 1.22)	.514	1.00 (0.73, 1.36)	.978	0.34 (0.11, 1.07)	.065
Diabetes mellitus						
No	Ref		Ref			
Yes	0.63 (0.4, 1.00)	.050	0.6 (0.37, 1.00)	.049	0.96 (0.35, 2.63)	.930
Dyslipidaemia						
No	Ref		Ref		Ref	
Yes	0.86 (0.64, 1.15)	.306	0.93 (0.68, 1.28)	.665	0.46 (0.20, 1.05)	.066
Abnormal haemoglobin						
No	Ref		n/a^b^		Ref	
Yes	0.48 (0.07, 3.42)	.463	n/a^b^		2.35 (0.32, 17.21)	.400
Abnormal total cholesterol						
No	Ref		Ref		Ref	
Yes	0.84 (0.60, 1.19)	.335	0.88 (0.61, 1.27)	.497	0.52 (0.18, 1.52)	.228
Abnormal triglyceride						
No	Ref		Ref		Ref	
Yes	0.85 (0.62, 1.17)	.320	0.83 (0.59, 1.17)	.287	1.21 (0.53, 2.78)	.651
Abnormal HDL-cholesterol						
No	Ref		Ref		Ref	
Yes	1.40 (1.09, 1.81)	.010	1.32 (1.00, 1.75)	.054	1.86 (1.03, 3.37)	.040
Abnormal LDL-cholesterol						
No	Ref		Ref		Ref	
Yes	1.37 (0.99, 1.89)	.061	1.44 (1.02, 2.03)	.040	0.91 (0.34, 2.42)	.855
Abnormal AST						
No	Ref		Ref		Ref	
Yes	0.91 (0.63, 1.33)	.629	0.94 (0.64, 1.40)	.775	0.79 (0.25, 2.46)	.680
Abnormal ALT						
No	Ref		Ref		Ref	
Yes	1.08 (0.81, 1.43)	.596	1.13 (0.84, 1.52)	.417	0.70 (0.30, 1.62)	.407

^a^Presented numbers indicate the adjusted values.

^b^Values are incalculable because of the small number of patients.

IBD, inflammatory bowel disease; UC, ulcerative colitis; CD, Crohn’s disease; HR, hazard ratio; FIT, faecal immunochemical test; BMI, body mass index; HDL, high-density lipoprotein; LDL, low-density lipoprotein; AST, aspartate aminotransferase; ALT, alanine aminotransferase.

In subgroup analyses based on disease subtypes, FIT positivity (HR 3.12, 95% CI: 2.60, 3.76, P <.001), male sex (HR 1.79, 95% CI: 1.45, 2.22, P <.001), and abnormal low-density lipoprotein (LDL)-cholesterol level (HR 1.44, 95% CI: 1.02, 2.03; P = .040) were associated with a greater risk of UC; however, the risk of UC decreased following an increase in BMI (HR 0.92, 95% CI: 0.89, 0.95, P <.001) and diabetes mellitus (HR 0.60, 95% CI: 0.37, 1.00, P = .049). In terms of CD, both FIT positivity (HR 2.04, 95% CI: 1.31, 3.19, P = .002) and abnormal HDL levels (HR 1.86, 95% CI: 1.03, 3.37, P = .040) exhibited an increased risk of developing CD (**Table 4**).

Furthermore, we performed an additional adjusted Cox analysis in participants who underwent colonoscopy after FIT (+) to evaluate the influence of colonoscopy results on IBD occurrence. However, positive colonoscopy results did not significantly influence the occurrence of IBD (**Table 5**), and only male sex (HR 1.38, 95% CI: 1.03, 1.84; P = .030) and BMI (HR 0.92, 95% CI: 0.88, 0.96; P <.001) were associated with the incidence of IBD.

**Table 5:** Predictive factors of developing IBD among participants with positive FIT who underwent colonoscopy.

	Univariable analysis		Multivariable analysis	
	HR (95% CI)	*P*-value	HR (95% CI)	*P*-value
Findings of colonoscopy				
Negative	Ref		Ref	
Positive	1.18 (0.91,1.53)	.2054	1.22 (0.93,1.59)	.151
Sex				
Female	Ref		Ref	
Male	1.33 (1.03,1.73)	.029	1.38 (1.03,1.84)	.0297
Age at screening, years				
50–59	2.55 (0.36,18.18)	.3519	3.04 (0.42,21.77)	.3519
60–69	2.05 (0.29,14.74)	.4753	2.36 (0.33,17.00)	.4753
70–79	1.81 (0.25,13.34)	.5611	1.99 (0.27,14.72)	.5611
≥80	Ref		Ref	
BMI	0.92 (0.88,0.96)	.0003	0.92 (0.88,0.96)	.0003
Smoking status				
No	Ref		Ref	
Yes	1.09 (0.78,1.52)	.6288	0.85 (0.59,1.23)	.3935
Alcohol drinking				
No	Ref		Ref	
Yes	1.10 (0.81,1.47)	.5471	1.03 (0.74,1.43)	.8673
Insurance type				
Medical aid	Ref		Ref	
National health insurance	0.37 (0.12,1.15)	.085	0.58 (0.24, 1.39)	.220
Hypertension				
No	Ref		Ref	
Yes	0.93 (0.62,1.41)	.7424	1.03 (0.68,1.56)	.8937
Diabetes				
No	Ref		Ref	
Yes	0.45 (0.21,0.95)	.0365	0.48 (0.22,1.02)	.0554
Dyslipidaemia				
No	Ref		Ref	
Yes	0.83 (0.62,1.11)	.2006	0.74 (0.49,1.13)	.1673
Abnormal total cholesterol				
No	Ref		Ref	
Yes	0.86 (0.63,1.18)	.3583	0.83 (0.51,1.36)	.458
Abnormal triglyceride				
No	Ref		Ref	
Yes	0.84 (0.59,1.21)	.3495	1.07 (0.69,1.68)	.7561
Abnormal HDL-cholesterol				
No	Ref		Ref.	
Yes	1.27 (0.90,1.8)	.1708	1.44 (0.99,2.09)	.0583
Abnormal LDL-cholesterol				
No	Ref		Ref	
Yes	1.08 (0.79,1.47)	.6458	1.53 (0.97,2.41)	.0688
Abnormal AST				
No	Ref		Ref	
Yes	0.72 (0.45,1.17)	.1841	0.74 (0.43,1.28)	.2842
Abnormal ALT				
No	Ref		Ref	
Yes	0.86 (0.61,1.22)	.4083	1.03 (0.68,1.54)	.8966

IBD, inflammatory bowel disease; FIT, faecal immunochemical test; HR, hazard ratio; BMI, body mass index; HDL, high-density lipoprotein; LDL, low-density lipoprotein; AST, aspartate aminotransferase; ALT, alanine aminotransferase.

### The incidence of IBD in the matched population

**Figure 4:**
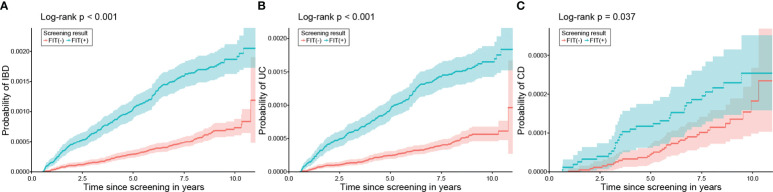
Probability of developing IBD, UC, and CD according to FIT results after PSM. In the matched population, patients with positive FIT results demonstrated a significantly higher risk of **(A)** IBD, **(B)** UC, and **(C)** CD than those with negative FIT results. IBD, inflammatory bowel disease; UC, ulcerative colitis; CD, Crohn’s disease; FIT, faecal immunochemical test; PSM, propensity score matching.

Given the considerable difference between the characteristics of the FIT (+) and FIT (−) groups, a 1:2 propensity score matching (PSM) was conducted to eliminate the difference. The demographic data and laboratory results were found to be comparable after PSM (**Table 6**). Kaplan–Meier analysis in this population also demonstrated an elevated risk of IBD in the FIT (+) group compared to that in the FIT (−) group (HR 2.85, 95% CI: 2.34, 3.48, P <.001). FIT positivity increased the risk of both incident UC (HR 3.17, 95% CI: 2.55, 3.94, P <.001) and CD (HR 1.68, 95% CI: 1.03, 2.74, P = .037) (**Figure 4**).

**Table 6:** Comparison of demographic data and laboratory results after a 1:2 PSM.

	Total cohort (n = 437,256)	FIT (−) (n = 291,504)	FIT (+) (n = 145,752)	SMD
Demographic data				
Sex				0.009
Male	247,192 (56.53)	165,225 (56.68)	81,967 (56.24)	
Female	190,064 (43.47)	126,279 (43.32)	63,785 (43.76)	
Age at screening, years				0.013
50–59	225,240 (51.51)	150,742 (51.71)	74,498 (51.11)	
60–69	149,965 (34.30)	99,600 (34.17)	50,365 (34.56)	
70–79	57,251 (13.09)	37,930 (13.01)	19,321 (13.26)	
≥80	4,800 (1.10)	3,232 (1.11)	1,568 (1.08)	
BMI, kg/m^2^	24.29 ± 3.04	24.29 ± 3.07	24.27 ± 2.98	0.009
Smoking status				0.001
No	367,244 (83.99)	244,880 (84.01)	122,364 (83.95)	
Yes	70,012 (16.01)	46,624 (15.99)	23,388 (16.05)	
Alcohol drinking				0.006
No	340,112 (77.78)	226,485 (77.70)	113,627 (77.96)	
Yes	97,144 (22.2)	65,019 (22.30)	32,125 (22.04)	
Insurance type				<0.001
Medical aid	2,231 (0.51)	1,485 (0.51)	746 (0.51)	
National health insurance	435,025 (99.49)	290,019 (99.49)	145,006 (99.49)	
Underlying comorbidity				
Hypertension				0.003
No	390,692 (89.35)	260,377 (89.32)	130,315 (89.41)	
Yes	46,564 (10.65)	31,127 (10.68)	15,437 (10.59)	
Diabetes mellitus				0.003
No	410,390 (93.86)	273,533 (93.84)	136,857 (93.90)	
Yes	26,866 (6.14)	17,971 (6.16)	8,895 (6.10)	
Dyslipidaemia				0.003
No	309,965 (70.89)	206,508 (70.84)	103,457 (70.98)	
Yes	127,291 (29.11)	84,996 (29.16)	42,295 (29.02)	
Laboratory results				
Haemoglobin, g/dL	13.81 ± 1.49	13.81 ± 1.49	13.82 ± 1.49	0.006
Total cholesterol, mg/dL	200.45 ± 40.87	200.45 ± 40.62	200.45 ± 41.34	0.002
Triglyceride, mg/dL	136.61 ± 88.60	136.55 ± 88.75	136.73 ± 88.30	0.002
HDL-cholesterol, mg/dL	54.04 ± 22.38	54.05 ± 20.73	54.01 ± 25.36	0.002
LDL-cholesterol, mg/dL	120.45 ± 50.33	120.42 ± 48.53	120.51 ± 53.75	0.002
AST, IU/L	28.00 ± 21.15	27.69 ± 21.25	28.63 ± 20.94	0.044
ALT, IU/L	26.50 ± 28.07	26.22 ± 24.99	27.04 ± 33.40	0.028

Data are presented as mean (SD) or number (%).

PSM, propensity score matching; FIT, faecal immunochemical test; SMD, standardised mean difference; BMI, body mass index; HDL, high-density lipoprotein; LDL, low-density lipoprotein; AST, aspartate aminotransferase; ALT, alanine aminotransferase.

## Discussion

Detection of faecal haemoglobin is a widely used method to screen for CRC, and FIT is recommended as a CRC screening test because it is considered to have higher diagnostic performance than the guaiac-based method (24). There is accumulating evidence linking positive faecal blood with diseases unrelated to CRC, indicating that this finding could reflect greater inflammation in the human body; this has been replicated in a number of studies (14, 25). Moreover, a previous investigation revealed that the risk of immune-mediated inflammatory disorders, particularly rheumatoid arthritis, is increased in patients with positive FIT results (17). In our study, by using the data of those who participated in a national CRC screening program, we identified a group of people who had positive FIT results but without evidence of apparent gastrointestinal bleeding. Importantly, we found that positive FIT results were independently associated with the occurrence of UC and CD, which was reproduced in the sensitivity analysis, indicating that FIT abnormalities in the general population could predict the onset of IBD.

The association between positive FIT and IBD occurrence observed in our study could be explained by the disruption of local bowel homeostasis and changes in the gut microbiome, which play significant roles in alterations of the immune landscape and imbalance of cytokines found in IBD (26, 27). First, the breakdown of localised gut homeostasis could shift the balance between pro- and anti-inflammatory mediators, contributing to the maintenance of intestinal mucosal integrity and inducing an abnormal systemic immune response (28). Second, interference of the balance in the gut microbiota is regarded as essential in inducing intestinal barrier damage and triggering inflammatory responses in IBD (29). Supporting this, *in vivo* studies have identified gut dysbiosis as a crucial component leveraging the development of IBD (30). Collectively, it could be assumed that abnormalities in the gut mucosa, defined as positive FIT results in our study, are associated with the evolution of IBD.

Predicting individuals at risk for IBD in the general population prior to disease development is highly challenging, although various risk factors for IBD have been identified (31). In our study, we demonstrated that FIT positivity confers an increased risk of IBD in the general population, even after excluding cases of IBD that were diagnosed after 6 months of screening. This finding implies that a positive FIT could be a preceding sign of IBD and could be applied to determine the high-risk population for developing IBD prior to the onset of overt disease. Notably, previous studies have reported that FIT can anticipate mucosal healing and has equivalent performance compared to faecal calprotectin (32, 33), which is the most widely adopted test for the identification of disease and quantification of inflammation in the bowel (34–36). In this context, those with a positive FIT result and symptoms that raise a suspicion of IBD might be candidates for regular screening for the presence of intestinal inflammation. Also, FIT could be advantageous as a screening test for IBD in the general population compared to faecal calprotectin, in terms of cost-effectiveness.

Our results revealed that 0.06% and 0.01% of participants were diagnosed with UC and CD, respectively, during the mean follow-up of 7.61 years. Although a large variation in the incidence rates of IBD has been reported in the literature, it is generally understood that the incidence of IBD is higher in European countries than in regions of the Eastern world (37). While the exact incidence of IBD in South Korea is not well understood, a previous study has shown that the estimated incidence of UC and CD is approximately 4/100,000 and 2/100,000, respectively, each year (38). Considering that IBD is relatively common in younger individuals, the incidence of IBD in our study (crude annual incidence rate, 0.98/10,000) was higher than that reported in the literature. Nonetheless, a higher proportion of participants developing UC than those with CD was also demonstrated, in agreement with current evidence. Notably, the discrepancies in UC and CD incidence compared with the previous study could be relevant to the study design. First, we identified those with positive FIT results and matched them with those showing a negative FIT result, which might have influenced the higher incidence of IBD. Second, the incidence of UC is relatively higher in the elderly than that of CD (39); because participants enrolled in the national CRC screening program were all aged >50 years, this would have resulted in a substantially higher UC annual incidence rate than CD.

The Cox proportional hazard analysis indicated that positive FIT, male sex, low BMI, presence of diabetes mellitus, and abnormal HDL cholesterol level were associated with the risk of subsequent IBD. The positive and negative relationship between age groups and female sex is consistent with the knowledge that IBD frequently occurs in men aged 30–40 years, with a decline in its incidence in the older population (40). In addition, the association of abnormalities in HDL cholesterol with IBD implies that changes in HDL cholesterol reflect alterations in the immune system, other than cardiovascular events (41). Also, BMI was inversely associated with the incidence of IBD. While it remains inconclusive whether low BMI confers a risk of developing IBD, population-based studies from Denmark have indicated an increase in IBD among participants with low BMI (42, 43), which may be partly explained as a consequence of chronic inflammation causing cachexia or representing a pre-clinical manifestation of IBD. Interestingly, we confirmed a negative correlation between diabetes mellitus and IBD, particularly UC. While there is a lack of studies exploring the association between diabetes mellitus and IBD, it has been reported that the use of metformin and dipeptidyl peptidase-4 inhibitors, which are the most frequently prescribed drugs for the management of diabetes mellitus in South Korea (44), could mitigate the risk of IBD in patients with diabetes mellitus (45, 46). In particular, as both drugs inhibit the activation of pro-inflammatory cytokines and improve insulin resistance, it is possible that they could decrease local inflammatory reactions in the intestine (47, 48). Therefore, the decreased risk of IBD in patients with diabetes mellitus could have been accounted for by the effects of these drugs, although we could not confirm the types of medication in The Korean National Cancer Screening Program (KNCSP) database. Lastly, the lack of association with smoking, a known environmental trigger for IBD, could be related to the fact that smoking status was categorised into current and non-current; additionally, the percentage of smokers was low, and the potential association of smoking may not have been evident because of other confounders (49).

An important strength of our study was that we demonstrated in a nationwide cohort that a positive FIT is associated with an increased the risk of future IBD development. However, our study has some limitations. First, although this was a large-scale study involving those who participated in a CRC screening program, we only had baseline information for the analysis of IBD incidence and the primary aim of CRC screening program was not to screen for IBD. Second, because the enrolled participants were exclusively >50 years of age, the risk of developing IBD among those with positive FIT results in the younger population could not be evaluated. Third, detailed colonoscopic findings could not be utilised in our analyses, and it could not be clarified whether a complete endoscopic examination was performed to exclude other potential causes. This was because full colonoscopy results were not provided because of the potential identification of individuals. Furthermore, whether other imaging tests were undertaken subsequently - such as magnetic resonance imaging small bowel study/a capsule study – could not be confirmed. Fourth, although various genetic and environmental factors, including dietary intake, could affect IBD incidence, such data are not available in the KNCSP and could not be analysed. Fifth, 14 patients were diagnosed with UC and CD simultaneously; although the number of patients was small, this may be an unclassified type of IBD showing overlapping features of UC and CD (50). However, the details of these patients could not be queried owing to the limitations of the National Health Insurance Sharing Service (NHIS). Finally, while measuring faecal calprotectin levels may be a useful strategy for early detection of IBD in patients with positive FIT and normal colonoscopy, these results were not available in the KNCSP database.

## Conclusion

In conclusion, by utilising the data of those who participated in the nationwide screening program for CRC, it was found that a positive FIT is associated with the onset of IBD in the general population. Abnormal FIT results could be a preceding sign of incident IBD, and regular screening may be beneficial, especially in patients with suspected symptoms of IBD.

## Data availability statement

The datasets presented in this article are not readily available because the datasets analysed in this study cannot be shared publicly because of national legislation for protection of personal information. However, data are available from the Korea National Health Insurance Sharing Service (contact via https://nhiss.nhis.or.kr, contact: +82-33-736-2432, 2433) for those authorised to access the confidential data. Requests to access the datasets should be directed to https://nhiss.nhis.or.kr.

## Ethics statement

The studies involving human participants were reviewed and approved by Ajou University Hospital (approval No. AJIRB-MED-EXP-20-479). Written informed consent for participation was not required for this study in accordance with the national legislation and the institutional requirements.

## Author contributions

SSA and C-KN: Conceptualisation. EL, SSA, and C-KN: Methodology. EL, GHL, SSA, and C-KN: Software. EL, SSA, and C-KN: Validation. EL and BP: Formal analysis. EL, SSA, and C-KN: Investigation. EL, GHL, SSA, and C-KN: Resources. EL: Data curation. GHL, SSA and C-KN: Writing—original draft preparation. EL, GHL, BP, SSA, and C-KN: Writing—review and editing. EL and C-KN: Visualisation. BP, SSA and C-KN: Supervision. EL, GHL, BP, SSA, and C-KN: Project administration. All authors contributed to the article and approved the submitted version.
