# Supplementary figures and images for: Positive faecal immunochemical test predicts the onset of inflammatory bowel disease: A nationwide, propensity score-matched study
Source: Front Immunol. 2023 Feb 13;14:1128736. doi: 10.3389/fimmu.2023.1128736 (PMC9968927; doi:10.3389/fimmu.2023.1128736)

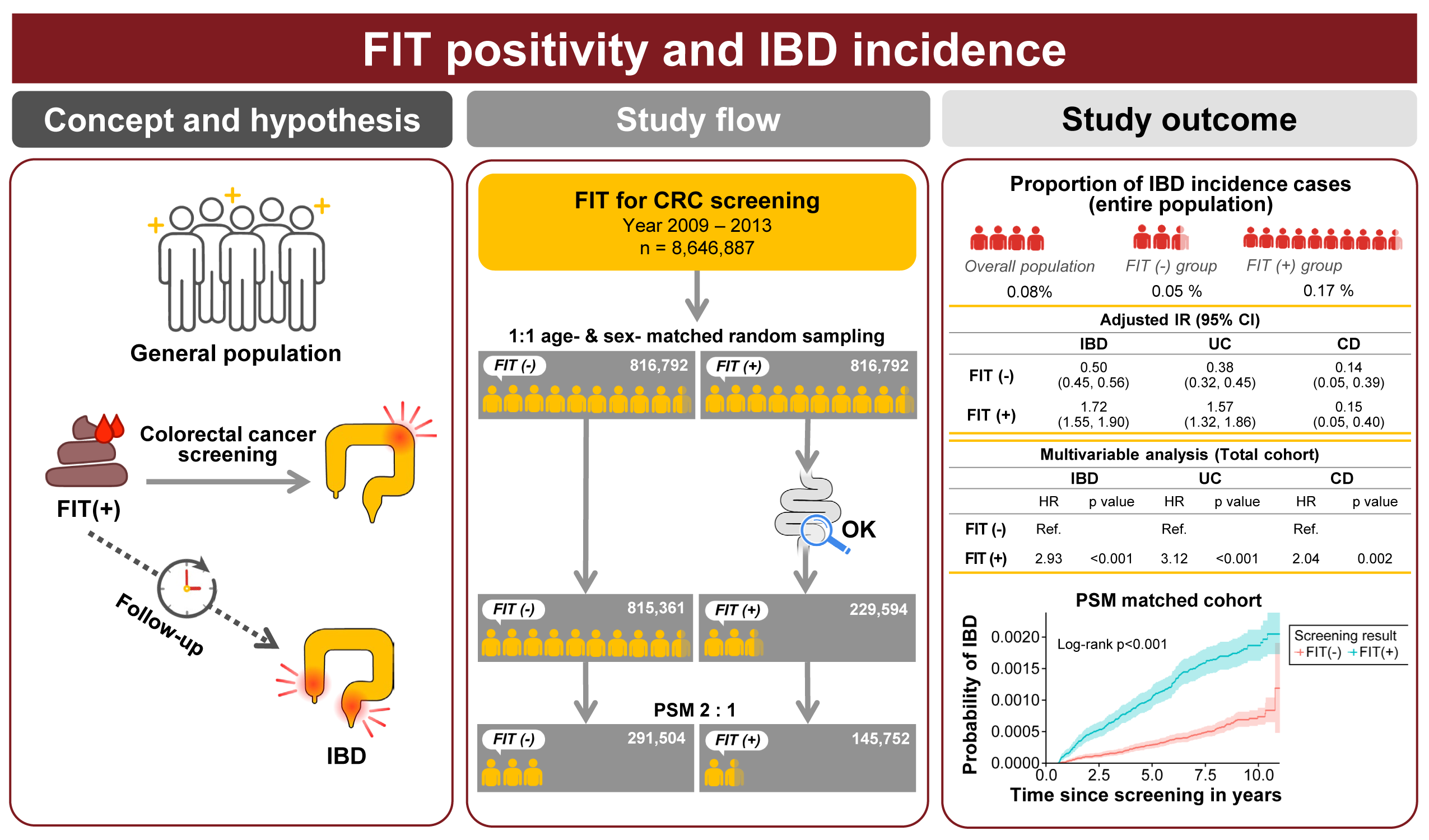

Supplement: Supplementary file 1 [file Image_1.tif]
